# Genetic investigation and diagnosis in adults with congenital heart disease with or without structural or neurodevelopmental comorbidity: a retrospective chart review

**DOI:** 10.3389/fgene.2024.1412806

**Published:** 2024-10-09

**Authors:** Moriah Edwards, Xue Zhang, Alexander R. Opotowsky, Nicole Brown, Amy R. Shikany, Kathryn Nicole Weaver

**Affiliations:** ^1^ Cincinnati Genetic Counseling Graduate Program, Cincinnati, OH, United States; ^2^ Division of Human Genetics, Cincinnati Children’s Hospital Medical Center, Cincinnati, OH, United States; ^3^ The Heart Institute, Cincinnati Children’s Hospital Medical Center, Cincinnati, OH, United States; ^4^ Department of Pediatrics, University of Cincinnati College of Medicine, Cincinnati, OH, United States

**Keywords:** adult congenital heart disease, extracardiac comorbidity, genetic testing, neurodevelopmental comorbidity, cardiology

## Abstract

**Introduction:**

Genetic evaluation is indicated for individuals with congenital heart disease (CHD), especially if extracardiac anomalies are also present. Timely recognition of genetic diagnoses can facilitate medical management and as well as provide assessment of reproductive risk. At least 20% of the pediatric population with CHD has a syndrome or genetic diagnosis. Further, at least 30% have extracardiac congenital malformations and/or neurodevelopmental differences (NDD), and this is known to increase the likelihood of a genetic/syndromic diagnosis. However, little is known regarding whether these statistics also apply to the current population of adults living with CHD, many of whom were born prior to currently available genetic testing.

**Methods:**

The primary aim of this study was to determine the prevalence of documented genetic and syndromic diagnoses in a cohort of adults with CHD followed by a dedicated adult CHD (ACHD) clinic. The secondary aims were to describe genetic testing and genetic referral patterns in this population and identify the presence of extracardiac comorbidities which are known to be indicative of an underlying genetic diagnosis in the pediatric CHD population. To answer these questions, we performed a retrospective chart review on a sample of adults with CHD (excluding those with isolated bicuspid aortic valve) seen at Cincinnati Children’s Hospital in the ACHD clinic between 2010–2021.

**Results:**

Among 233 adult CHD patients, 36 (14%) had a documented genetic or syndromic diagnosis but only 29 (13.7%) had received genetic testing, while 27 (11.6%) had received genetic referrals. Furthermore, of 170 patients without any documented genetics related care (defined as genetic testing, genetic referrals, or genetic diagnosis), 35 (20%) had at least one congenital and/or neurodevelopmental comorbidity. Factors associated with individuals having received genetics related care included younger age (<40), male sex, and presence of extracardiac comorbidities.

**Discussion:**

Our results indicate important gaps in genetics-related care for adults living with CHD. The subset of our cohort with congenital and/or neurodevelopmental comorbidities who received no genetic-related care, represent a population of adults with CHD who may have unrecognized genetic diagnoses.

## 1 Introduction

Congenital heart disease (CHD) is one of the most prevalent birth defects, occurring in approximately 1 out of every 100 live births (Mitchell et al., 1971; Van Der Linde et al., 2011; Marino et al., 2012). Medical advances have decreased mortality rates in individuals with CHD. As many as 90% of children with CHD now reach adulthood (Khairy et al., 2010; Ntiloudi et al., 2016; Billotte et al., 2021), and adults living with CHD outnumber children with CHD (Pierpont et al., 2018).

The pediatric CHD patient population has been well-studied with respect to extracardiac comorbidities and neurodevelopmental disorders and have increased rates of both. Approximately 20%–30% of the pediatric CHD population have congenital extracardiac abnormalities (Massin et al., 2007; Ferencz et al., 1989; Helm and Ware, 2024). It has been shown that multisystem involvement increases the likelihood of genetic diagnoses in children with CHD (Massin et al., 2007; Bracher et al., 2017; Cohen et al., 2013; Shikany et al., 2020) and in general, patients with genetic syndromes are more likely to have extracardiac comorbidities compared with non-syndromic patients (Bracher et al., 2017; Khanna et al., 2019). However, not all patients presenting with CHD and coexisting extracardiac diagnoses have a specific, identifiable genetic syndrome, and individuals with a syndromic diagnosis may present with apparently isolated CHD and no other comorbidities. Therefore, isolated CHD and apparent lack of syndromic diagnosis does not exclude the possibility of genetic etiology for CHD (Massin et al., 2007; Bracher et al., 2017; Hoang et al., 2018).

The testing currently available and offered to infants with CHD was developed within the past one to 2 decades. With development of chromosomal microarray and next-generation sequencing (NGS) genetic testing technology in the early 2000s (Rauch et al., 2004; Slatko et al., 2018), diagnostic capabilities of genetic testing have greatly improved as has recognition of the utility of genetic testing for individuals with CHD. Clinical genetic evaluation and broad genetic testing that is now readily accessible (genome and exome sequencing) were essentially unavailable for adult patients at the time of their cardiac diagnosis (Lalani, 2020; Zaidi and Brueckner, 2017). We hypothesized that the prevalence of documented (recognized) genetic diagnoses in an ACHD population would be lower than pediatric populations and that the population of adults living with CHD would have relatively low rates of genetic testing and referral compared with what is typically provided to individuals born in more recent years. Identifying and defining gaps in genetics-related care for the ACHD population are important steps in improving referral and diagnosis rates and ultimately improving patient care. Therefore, we performed a retrospective study to identify the prevalence of three elements of genetic-related care (syndromic/genetic diagnoses, genetic referral, genetic testing) in an adult CHD population, and identify patient traits and characteristics, including congenital and neurocognitive comorbidities, that are associated with having received genetic-related care and/or are indicators of a population that would benefit from genetics care.

## 2 Materials and methods

### 2.1 Selection and description of participants

A retrospective chart review was performed with approval from the Cincinnati Children’s Hospital Medical Center (CCHMC) Institutional Review Board for adults with CHD who received care at between 1/1/2010 and 10/31/2021. We queried the electronic medical record (EMR) to identify adult patients (≥18 years at time of query) who had a CCHMC cardiology visit of any of the 11 EMR visit types used in the ACHD Clinic (see Supplementary Table S1). Visit types in the EMR are digital templates designated by 3- to 5-digit codes that are designed for specific clinic use. In the ACHD clinics, this includes templates for new patients, follow-up visits, and Fontan clinic-specific scenarios. A total of 2,275 unique patients resulted from the EMR query. We determined that a minimum of 108 patients should be included for appropriate statistical power. This was calculated based on our primary hypothesis that the proportion of genetic diagnoses in adults with CHD differs from that in pediatric patients with CHD. Previous studies have suggested that the proportion in pediatric patients is 20%. In our adult cohort, the proportion is expected to be 10%. A total of 108 subjects will allow us to detect the difference with 80% power when the type I error rate is set at 0.05. Using the Microsoft Excel (Version 2201) randomizing tool, we randomly sampled the queried list of patients to select approximately equal number by age group (<40 years of age and ≥40 years of age) and sex. We selected 325 cases for further review.

### 2.2 Inclusion/exclusion criteria

For 325 randomly selected cases, we verified patient age at most recent ACHD clinic visit, the presence of a personal CHD diagnosis, and adherence to all other inclusion and exclusion criteria. Patients were excluded if they were less than 18 years old at time of most recent ACHD clinic visit (8 patients); if they did not have a primary diagnosis of CHD (31 patients); or if they were not seen in the ACHD clinic (29 patients). Patients with isolated bicuspid aortic valve and/or thoracic aortic aneurysm and patients with aortic dilation were also excluded (24 patients). Of note, patients with Noonan syndrome and Marfan/Loeys Dietz syndromes are seen in separate dedicated clinics at CCHMC and therefore were excluded by our search criteria. Of the 325 initially selected cases, 92 were excluded. The final cohort consisted of 233 patients (demographics in Table 1).

**TABLE 1:** Characteristics of adults with CHD. N = 233 except where data was missing from chart and in “Genetic diagnosis,” where 4 patients were excluded due to syndromic diagnoses not traditionally associated with CHD. Excluded patients were counted in “Genetic diagnosis by type” under “Other.”

Characteristics of adults with CHD.	N	%
Age (y) (n = 233)		
18–40	132	43.4
40+	101	56.7
Sex (n = 233)		
Male	95	40.8
Female	138	59.2
Race(n = 230)		
White	200	87.0
Black	23	10.0
Other	4	1.7
Mixed race (2 or more)	3	1.3
Cardiac lesion (n = 233)		
Tetralogy of Fallot or DORV or pulmonary atresia	46	19.7
Left-sided obstructive lesions	35	15.0
AV septal defect	19	8.2
Valvar pulmonary stenosis	17	7.3
D-TGA or physiologically corrected TGA (systemic right ventricle) or s/p arterial switch (systemic left ventricle)	16	6.9
Ebstein/Uhl anomaly	8	3.4
Heterotaxy spectrum	4	1.7
Simple shunt lesions	60	25.6
Miscellaneous/other	16	6.9
Acquired comorbidities (n = 233)		
None	126	54.3
At least one	106	45.7
Congenital + ND comorbidities (n = 228)		
Only congenital comorbidity	45	19.5
Only ND comorbidity	13	5.6
Both congenital and ND comorbidity	21	9.1
None	152	65.8
Family history of CHD (n = 233)		
No	202	86.7
Yes	31	13.3
History of any CHD-related genetic testing (n = 211)		
No	182	86.3
Yes	29	13.7
History of genetic referral (n = 233)		
No	206	88.4
Yes	27	11.6
Genetic diagnosis (n = 233)		
No	197	84.5
Yes	32	13.7
Yes, likely unrelated to CHD	4	1.7
Genetic diagnosis by type (n = 36)		
Down	17	47.2
22q11 (DiGeorge, CATCH 22, VCF)	7	19.4
Turner	2	5.6
Williams	2	5.6
CHARGE	2	5.6
Other	5	13.9
Not specified	1	2.8

### 2.3 Chart review methods

Data collected from the EMR were recorded in a REDCap database (see Supplementary Material). We collected information about established genetic diagnoses, history of genetic testing, and history of genetic referrals. We also collected detailed data on cardiac history and lesion type, and extracardiac comorbidities (including acquired conditions and congenital/structural anomalies).

Patients were considered to have a genetic or syndromic diagnosis if they had a pathogenic/likely pathogenic genetic test result, or a clinical diagnosis of a syndrome (e.g., Down syndrome) documented in their medical record, even if confirmatory genetic testing was not conducted or if that record was missing. A patient was defined as having had genetic testing if records of genetic testing were available or if genetic testing was specifically referenced in clinical notes, even if the original report was missing. We recorded genetic referrals when the patient had documentation of a clinical genetic evaluation or if a genetics referral request had been placed in the EMR, even if this had not yet been completed.

CHD diagnoses were classified by dominant CHD type (i.e., the most severe diagnosis, in terms of impact on clinical status). If dominant CHD type was not clearly documented in the patient’s chart, the chart was reviewed by an ACHD clinic cardiologist (AO) to clarify the dominant CHD type.

We collected information about each patient’s neurodevelopmental status, acquired medical comorbidities (e.g., gastrointestinal reflux disease, hypertension), and congenital conditions (e.g., craniofacial dysmorphism, cleft palate). We assessed neurodevelopmental status based on documentation of neurocognitive disorders (e.g., intellectual disability) and highest level of school completed. We distinguished neurodevelopmental comorbidities from neurological comorbidities (e.g., stroke, seizures, migraines) and psychiatric comorbidities (e.g., depression, anxiety, schizophrenia). Neurodevelopmental comorbidities included attention deficit hyperactivity disorder (ADHD), cognitive impairment/developmental delay, and autism spectrum disorder.

We documented whether each patient had any biological children, any reported family history of CHD in first, second, and/or third-degree relative(s), and if affected relatives had extracardiac or neurodevelopmental comorbidities. Family history variables were collapsed into ‘family history’ or ‘no family history’ for analysis.

### 2.4 Statistical analyses

Prior to analysis, the quality and distribution of the data were examined. Demographics and clinical characteristics of the cohort were described using frequencies (proportions). To compare the genetic diagnosis rate of our cohort to that of previously reported pediatric cohorts, we conducted a one-sample proportion test. The associations of genetic diagnoses, referral, and testing with demographics and clinical characteristics were tested using Fisher’s exact tests. All analyses were performed using SAS 9.4 (company, Cary, NC). A p-value ≤ 0.05 was used to indicate the statistical significance.

## 3 Results

Demographics and clinical characteristics of the cohort are summarized in Table 1. The majority (86.7%) of patients were White and approximately 59% were female. The cohort was roughly evenly split between those younger and older than 40 years at time of chart review. The two most prevalent types of cardiac lesions were left-sided obstructive lesions (19.7%) and tetralogy of Fallot (15%).

Genetic or syndromic diagnoses were documented in 36 patients (36/233, 15%). However, we discovered four patients with syndromic diagnoses that are not typically associated with CHD, including Long QT syndrome, Charcot-Marie-Tooth syndrome, and hypermobile Ehlers Danlos syndrome. Therefore, these were excluded from the group with genetic syndromes and not included in the nonsyndromic group. Down syndrome made up 47% of the syndromic diagnoses (17/36), followed by 22q11.2 microdeletion syndrome (7/36, 19%).

Twenty-nine patients (29/211, 13.7%) had documented genetic testing. Of note, the 36 patients with genetic/syndromic diagnoses only encompassed 10 of the 29 with documented pathogenic findings on genetic testing, indicating that the majority of the 36 had clinical diagnoses. Of those with genetic testing, the majority (21/29, 72.4%) had only one test conducted, with FISH being the most common single test performed. Twelve patients had FISH testing which confirmed diagnosis of 22q11.2 microdeletion syndrome in 5. Six patients had microarray, all of which had normal results. Eight patients received single gene and/or multigene panel testing, which produced a diagnostic result in a single patient. Four patients had single gene or multigene panel testing in tandem with at least one other genetic test. No patients had exome or genome sequencing (Supplementary Table S2).

Twenty-seven patients (27/233, 11.6%) received a referral to a geneticist and/or genetic counselor, with a consult completed in 19/27 (70%). The remainder of referrals were pending appointments (n = 5) or missing data (n = 2) about referral status. Of the 27 with referrals, 11 had a genetic/syndromic diagnosis.

We tested the associations of demographics and clinical characteristics with the likelihood of a genetic diagnosis, genetic referral, or genetic testing (Table 2). Younger patients (<40 years of age) were more likely to have genetic testing on record (*p* = 0.048) and more likely to have a documented genetic/syndromic diagnosis (*p* = 0.003). Younger patients were more likely to have received a genetics referral (15.1% vs 6.9%), though this difference did not reach statistical significance. Cardiac lesion type was associated with presence of a genetic diagnosis (*p* < 0.001). While there was not a significant association detected between lesion type and genetic testing, a higher percentage of patients with tetralogy of Fallot received genetic testing compared to other cardiac lesion groups (29.3% versus <25%).

**TABLE 2:** Characteristics of adults with CHD by genetic referral, testing, and diagnosis. N is not equal across all categories due to missing data except in “Number of Relatives with CHD”, which contains only the patients with a family history of CHD, and in “Genetic Diagnosis,” where 4 patients were excluded due to syndromic diagnoses not traditionally associated with CHD.

Characteristics of adults with CHD by genetic referral, testing, and diagnosis status												
All traits	n	Genetic referral		p	n	Genetic testing		p	n	Genetic diagnosis		p
		Yes	No			Yes	No			Yes	No	
Age	233	N (%)	N (%)	0.06	211	N (%)	N (%)	0.048	229	N (%)	N (%)	0.003
18–40		20 (15.5)	112 (84.9)			21 (18.6)	92 (81.4)			26 (20)	104 (80)	
>40		7 (6.9)	94 (93.1)			8 (8.2)	90 (91.8)			6 (6.1)	93 (93.9)	
Sex	233			0.06	211			0.84	229			0.17
Male		16 (16.8)	79 (83.2)			13 (14.6)	76 (85.4)			9 (9.7)	84 (90.3)	
Female		11 (8)	127 (92)			16 (13.1)	106 (86.9)			23 (16.9)	113 (83.1)	
Race	230			0.38	209			0.84	226			
White		22 (11)	178 (89)			25 (13.9)	115 (86.1)			29 (14.8)	167 (85.2)	0.84
Black		5 (21.7)	18 (78.3)			4 (18.2)	18 (81.8)			2 (8.7)	21 (91.3)	
Other		0	7 (100)			0 (0)	7 (100			0 (0)	7 (100)	
Cardiac lesion	233			0.37	211			0.07	229			<0.001
ToF, DORV, PA		9 (19.6)	37 (80.4)			12 (29.3)	29 (70.7)			9 (19.6)	37 (80.4)	
Left-side obstructive		4 (11.4)	31 (88.6)			3 (9.1)	30 (90.9)			3 (8.6)	32 (91.4)	
AV septal		0	19 (100)			0	10 (100)			10 (52.6)	9 (47.4)	
Valvar PS		1 (5.9)	16 (94.2)			0	17 (100)			0	17 (100)	
D-TGA, phys. Corrected TGA, s/p arterial switch		3 (18.8)	13 (81.3)			1 (6.3)	15 (93.7)			0	16 (100	
SV Fontan or complex of SV unrepaired cyanotic		2 (16.7)	10 (83.3)			2 (16.7)	10 (83.3)			0	12 (100)	
Ebstein/Uhl		0	8 (100)			0	8 (100)			0	8 (100)	
Heterotaxy		1 (25)	3 (75)			1 (25)	3 (75)			0	4 (100)	
Simple shunt		6 (Helm and Ware, 2024)	54 (90)			7 (12.7)	48 (87.3)			7 (12.5)	49 (87.5)	
Miscellaneous/other		1 (6.3)	15 (93.8)			3 (20)	12 (80)					
Congenital/ND comorbidities	228			<0.001	208			<0.001	229			<0.001
Congenital		5 (11.1)	40 (88.9)			5 (13.9)	31 (86.1)			11 (24.2)	34 (75.6)	
ND		4 (30.8)	9 (69.2)			5 (41.7)	7 (58.3)			1 (7.7)	12 (92.3)	
Congenital + ND		8 (38.1)	13 (61.9)			10 (83.3)	2 (16.7)			16 (80)	4 (20)	
None		9 (5.9)	143 (94.1)			9 (6)	141 (94)			2 (1.3)	147 (98.7)	
Fam history CHD	233			0.22	211				30			0.58
Yes		6 (19.4)	21 (84)			7 (25)	21 (75)			5 (20.8)	19 (79.2)	
No		21 (10.4)	4 (66.7)			22 (12)	161 (88)			0	6 (100)	

**FIGURE 1:**
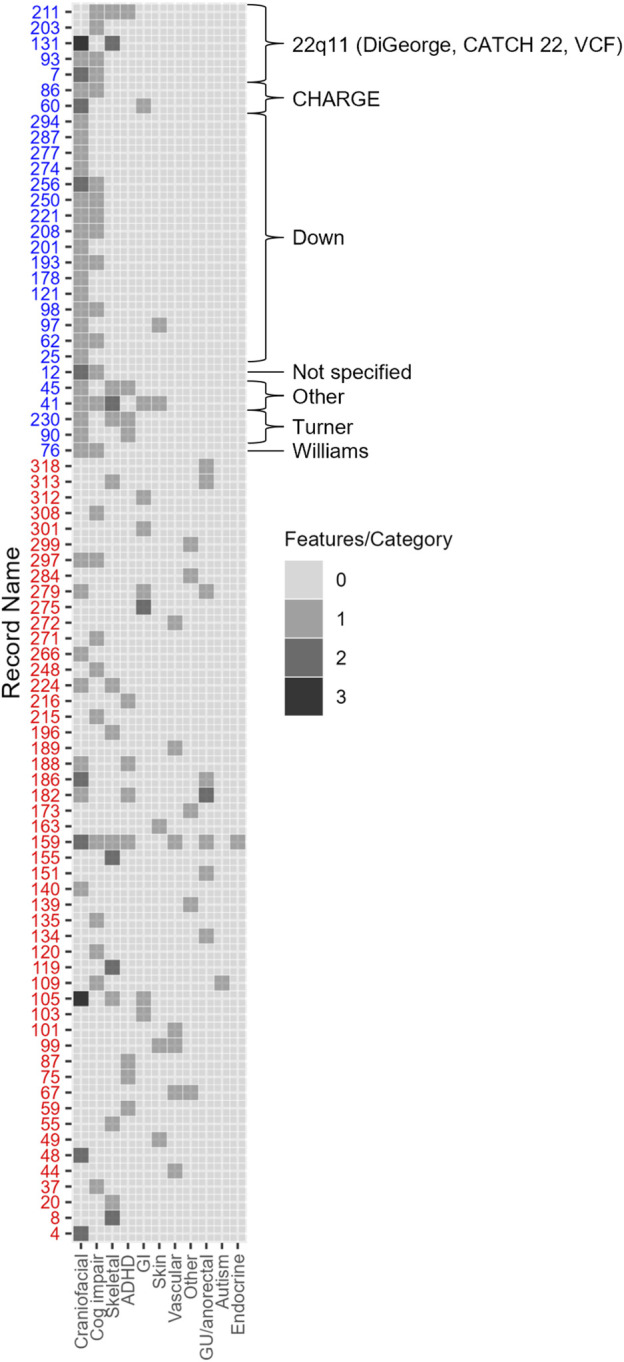
79 adults with CHD had extracardiac and/or neurodevelopmental comorbidities. Each row represents one patient with syndromic cases noted by blue text. Craniofacial comorbidities include craniofacial dysmorphism and anomalies of the brain, ear, nose, throat, palate, and eyes. Vascular comorbidities include anomalies of the lungs, lymphatic system, and arteriovenous malformations. GI and abdominal comorbidities include anomalies of the abdominal wall, kidney, spleen, pancreas, liver, and gall bladder. Number of comorbidities in each category was tabulated from each case from the data entry form (Supplementary Material) and boxes are shaded according to the number of manifestations present in each category shown on the *x*-axis.

Approximately 34% (79/228) of the cohort had at least one congenital and/or neurodevelopmental comorbidity (Figure 1). These patients were more likely to have genetic/syndromic diagnosis (*p* < 0.001), referral (*p* < 0.001), or testing (*p* < 0.001) if at least one congenital and/or neurodevelopmental comorbidity were present (Table 2). Increasing number of congenital and neurodevelopmental comorbidities is also associated with genetic referral (*p* < 0.001) (Table 3). By body system, those with a history of craniofacial (*p* < 0.001), skeletal (*p* < 0.001), or genitourinary and anorectal (*p* = 0.048) abnormalities were more likely to have genetic referrals on record. Of the neurodevelopmental comorbidities, ADHD and cognitive impairment were associated with genetic referral (*p* < 0.05). Of 170 patients who had no genetic testing, referral, or syndromic diagnosis, 35 (20%) had at least one congenital and/or neurodevelopmental comorbidity.

**TABLE 3:** Types and amounts of congenital and neurodevelopmental comorbities by referral status. N is not equal across all categories due to missing data.

Types and numbers of congenital and neurodevelopmental comorbidities by referral status							
		N	Genetic referral		No genetic referral		*p*-value
Congenital Comorbidities by Type			Number	%	Number	%	
	Craniofacial	231	28	71.2	11	28.1	*p* < 0.001
	Vascular systems	231	6	85.7	1	14.3	*p* = 0.57
	Endocrine	233	0	0	1	100.0	*p* = 0.12
	Gastrointestinal	231	6	75	2	25.0	*p* = 0.22
	Skeletal/Limb	233	7	46.7	8	53.3	*p* < .001
	Genitourinary/anorectal	231	5	62.5	3	37.5	*p* = 0.048
	Skin	233	4	66.7	2	33.3	*p* = 0.14
Number of Congenital Comorbidities		231	Number	%	Number	%	*p* < .0001
	0		13	7.9	152	92.1	
	1		4	8.5	43	91.5	
	2		3	27.3	8	72.3	
	3		3	60	2	40.0	
	4		2	100	0	0.0	
	5		1	100	0	0.0	
Neurodevelopmental Comorbidities by Type			Number	%	Number	%	
	ADHD	233	4	36.4	7	63.6	*p* = 0.026
	Cognitive impairment/developmental delay	233	10	40	15	60.0	*p* < 0.001
	Autism spectrum disorder	233	1	100	0	0.0	*p* = 0.11
Number of Neurodevelopmental Comorbidities		232	Number	%	Number	%	*p* < 0.001
	0		14	7.1	184	92.9	
	1		9	29	22	71.0	
	2		3	100	0	0.0	

Of the cohort, thirty-one patients (31/233, 13.3%) had a documented family history of CHD. Positive family history of CHD was not significantly associated with genetic testing, genetic referral, or genetic diagnosis, although a higher proportion of patients with a positive family history had genetic testing compared with those who had no family history (7/28, 25% versus 22/183, 12%). As expected, lesions with higher heritability (Oyen et al., 2009) had the highest incidence of positive family history (Table 4), including shunt lesions (25.8%), tetralogy of Fallot (16%), and left-sided obstructive lesions (16%) although this result did not reach statistical significance (*p* = 0.53). Five patients were reported to have a biological child with CHD which included 1 vascular ring, 1 ASD, 1 VSD and 1 unknown.

**TABLE 4:** Family history by cardiac lesion type.

Family history by cardiac lesion type					
	Family history		No family history		*p*-value
Cardiac lesion (n = 233)	Number	%	Number	%	0.53
Tetralogy of Fallot or DORV or pulmonary atresia	5	16.1	41	20.3	
Left-sided obstructive lesions	5	16.3	30	14.9	
AV septal defect	1	3.2	18	8.9	
Valvar pulmonary stenosis	2	6.5	15	7.4	
D-TGA or physiologically corrected TGA (systemic right ventricle) or s/p arterial switch (systemic left ventricle)	1	3.2	15	7.4	
Single ventricle (SV) Fontan-spectrum or Complex or SV unrepaired cyanotic (e.g., ToF/PA/MAPCA, mixing lesions s/p palliation)	2	6.4	10	5	
Ebstein/Uhl anomaly	3	9.7	5	2.5	
Heterotaxy spectrum	0	0	4	2	
Simple shunt lesions	8	25.8	52	25.8	
Miscellaneous/other	4	12.9	12	5.9	

## 4 Discussion

There is widespread agreement that adults with CHD need multidisciplinary care in light of increased survival from childhood to adulthood and the spectrum of extracardiac and neurodevelopmental diagnoses in the CHD population (Bracher et al., 2017; Pierpont et al., 2007; Stout et al., 2018). Currently, there are no established guidelines governing referral of adult patients with extracardiac anomalies or neurocognitive diagnoses to outside specialties such as genetics (Cohen et al., 2013). Our study demonstrated that there are gaps in genetics-related care for adults with CHD at our institution. The overall prevalence of a genetic/syndromic diagnosis in our cohort was 14%, which is similar to what has been reported in incompletely sequenced/tested pediatric cohorts such as the initial description of the Pediatric Cardiac Genomics Consortium (PCGC) cohort which reported genetic/syndromic diagnosis in 11% of ∼9,700 children (Hoang et al., 2018). Rates of trisomy 21 and deletion 22q11 syndrome, two genetic syndromes with well-established associations to CHD, were also similar between our adult CHD cohort and the 2018 PCGC cohort description (47% adult vs. 38% PCGC for Trisomy 21, and 19% adult vs. 24% PCGC for deletion 22q11). This, plus additional prior similarly described cohorts, suggests that the incidence of obvious genetic/syndromic diagnoses in CHD cohorts is around 10% (Massin et al., 2007; Zaidi and Brueckner, 2017; Egbe et al., 2014; Meberg et al., 2007).

In our study, factors associated with an adult having received genetics-related care (defined as genetic referral or testing) included younger age (18–40 years), male sex, and presence of extracardiac anomalies or neurodevelopmental diagnoses. The higher care rate in younger individuals likely reflects the increased availability of genetic testing and awareness of genetic contributions to CHD in more recent years. As expected, our data shows an association between cardiac lesion type and genetic diagnosis which we suspect was largely driven by the high rate of individuals with trisomy 21 and atrioventricular canal in our cohort. The types of genetic testing completed in our cohort reflect the technologies that were clinically available at the time, with karyotypes, microarray, and FISH being the most common. Microarray became available in our institution in 2008, only 16 years ago. Interestingly, documented family history of CHD was not found to have a statistically significant association with genetic referral or testing although a higher percentage of those with family history were referred to genetics compared to those without family history (19.4% versus 10.4%). Assessment of a larger cohort may provide further clarity. It is also worth noting that family history of CHD might not be fully assessed or documented by the managing cardiologist.

Our study, which found 79/233 (33%) of adults with CHD also had at least one extracardiac congenital abnormality and/or NDD, agrees with others that estimate 10%–50% of CHD patients have extracardiac comorbidities (Massin et al., 2007; Bracher et al., 2017; Hoang et al., 2018; Egbe et al., 2014). However, despite the general likelihood of our cohort to have received genetic-related care in the presence of congenital and/or neurodevelopmental comorbidities, there were many patients (35/233, 15%) with non-isolated CHD who have never received any genetic referral or testing. A genetic diagnosis is more likely to be identified in patients with CHD who have one or more extracardiac comorbidities and/or NDD (Bracher et al., 2017; Shikany et al., 2020). Therefore, these individuals may represent a population of adults with CHD who harbor unrecognized genetic diagnoses.

Our results suggest that adults with CHD may benefit from a more consistent and comprehensive genetic referral and testing strategy that utilizes available resources and newer technology and that aligns with current pediatric practices. This type of strategy should include analysis of both chromosomal and genetic sequence variants to minimize limitations of a less broad genetic testing approach. This study highlights the need to better utilize the available genetics expertise when evaluating genetic etiology of CHD. The utility of genetic testing for adults with CHD includes improved management and more accurate recurrence risk information. Even patients in our cohort who have received any historical genetic testing may be under-evaluated from a genetics standpoint in the current era. This is particularly true for the older ACHD population, who are less likely to receive any genetic-related care. Research has focused on the lack of knowledge or comprehension about cardiac lesion type, inheritance, pregnancy risks, and recurrence risks in adolescent patients, adult patients, and parents of patients, with emphasis that risk counseling has a positive impact on patient psychosocial functioning, medical management, and reproductive decision-making (Van Deyk et al., 2010; Van Engelen et al., 2011; Van Engelen et al., 2013; Blue et al., 2015; Shikany et al., 2019).

### 4.1 Conclusions

Medical professionals providing care to adults with CHD should be aware that this population (particularly older adults, >40 years) is highly under evaluated and under-counseled with respect to genetics. All adults with CHD who have any non-cardiac congenital and/or neurodevelopmental comorbidities or family history of CHD should be referred for a formal genetics assessment. As our understanding of genetic etiologies for CHD continues to evolve, even adults with a history of limited genetic testing for CHD are advised to obtain updated genetic evaluation and to be offered more comprehensive genetic testing when appropriate.

### 4.2 Limitations

A retrospective chart review has expected limitations, such as survival bias, missing records, potential inconsistencies in documentation between providers, and misclassification bias, as well accrual of cases from a single tertiary care center. Additionally, cardiologists may not reliably observe or record all congenital and/or neurodevelopmental comorbidities, prenatal history, or family history, in comparison to common practice among geneticists and therefore this could introduce bias in our assessment of presence of extracardiac comorbidities. Genetic testing referral and testing rates may be under ascertained due to documentation inconsistency and/or noncompliance/lack of uptake of recommended evaluations. Statistical significance might not have been achieved in some calculations due to the study being underpowered for certain comparisons. Finally, it is important to acknowledge that due to the presence of specialty clinics within our institution for patients with Marfan and related syndromes, and for patients with Rasopathies, these patient populations are under-represented in our cohort.

### 4.3 Future research

With the current data, further investigation into the specific comorbidities and their genetic testing and/or evaluation could be conducted. Future studies should include prospective studies for adult patients with CHD, comparative studies of genetic-related care with patients from a cardiovascular genetics clinic, and further investigation of adult patient comorbidities correlated with results of comprehensive genetic testing and evaluation.

## Data Availability

The datasets presented in this article are not readily available because individual level patient clinical information could compromise anonymity. Requests to access the datasets should be directed to kathryn.weaver@cchmc.org.

## Abbreviations

ACHD, Adult (or adults with) congenital heart disease; ADHD, Attention deficit hyperactivity disorder; CCHMC, Cincinnati Children’s Hospital Medical Center; CHD, Congenital heart disease; EMR, Electronic medical record; NDD, Neurodevelopmental disorders; WES, Whole exome sequencing; WGS, Whole genome sequencing
