# Supplementary material for: Genetic investigation and diagnosis in adults with congenital heart disease with or without structural or neurodevelopmental comorbidity: a retrospective chart review
Source: Front Genet. 2024 Oct 9;15:1412806. doi: 10.3389/fgene.2024.1412806 (PMC11496128; doi:10.3389/fgene.2024.1412806)
Supplement: Supplementary file 1 [file DataSheet1.PDF]

Codebook

Data Dictionary Codebook

02/17/2022 7:42pm

Collapse all instruments

#	Variable / Field Name	Field Label<br>*Field Note*	Field Attributes (Field Type, Validation, Choices, Calculations, etc.)
Instrument: **Demographics** (demographics)			Collapse
1	record_name	Study ID	text
2	ineligible	This patient was determined to be ineligible for the study.	radio<br>1 Yes
3	ineligible_describe<br>Show the field ONLY if:<br>[ineligible] = '1'	State reason for ineligibility.	notes
4	last_name	Last name	text, Required, Identifier
5	first_name	First name	text, Required, Identifier
6	mrn	MRN	text (number), Required, Identifier
7	dob	Date of birth<br>*MM-DD-YYYY*	text (date_mdy, Max: 2003-07-01), Required, Identifier
8	sex	Sex	dropdown, Required<br>1 Male<br>2 Female<br>3 Other
9	race_ethnicity	Race/Ethnicity	checkbox<br>1 race_ethnicity___1 White (non-Hispanic)<br>2 race_ethnicity___2 African American<br>3 race_ethnicity___3 Asian<br>4 race_ethnicity___4 American Indian or Alaskan native<br>5 race_ethnicity___5 Native Hawaiian<br>6 race_ethnicity___6 Hispanic, White<br>7 race_ethnicity___7 Hispanic, Non-White<br>8 race_ethnicity___8 Mixed race<br>9 race_ethnicity___9 Unknown<br>10 race_ethnicity___10 Other<br>444 race_ethnicity___444 Missing
10	education_level	Highest grade level completed	dropdown<br>1 Some high school<br>2 High school diploma<br>3 GED<br>4 Some college credit, no degree<br>5 Associates degree (AA, AS)<br>6 Bachelor's degree (BA. BS)<br>7 Master's degree (MA, MS, MEng, MEd, MSW, MBA)<br>8 Professional or doctorate degree (MD, DDS, DVM, LLB, JD, PhD, EdD)<br>444 Missing
11	guardian_status	Guardian status	dropdown<br>1 Own decision maker<br>2 Family guardian<br>3 Court appointed/state guardian<br>444 Missing

12	marital_status	Marital status	dropdown<br>1 Married<br>2 Widowed<br>3 Divorced<br>4 Separated<br>5 Never married<br>6 Never married, living with partner<br>444 Missing
13	vital_status	Last known vital status	dropdown<br>0 Deceased<br>1 Alive<br>444 Missing
14	dov_achd	Section Header: *Please enter the following data from most recent ACHD Clinic visit:*<br>Date of most recent ACHD Clinic visit<br>*MM-DD-YYYY*	text (date_mdy, Min: 2010-01-01), Required
15	age_achd	Age at most recent ACHD Clinic visit	calc<br>Calculation: rounddown(datediff([dob],[dov_achd],"y"))
16	height_achd	Height (cm)	text (number_1dp)
17	weight_achd	Weight (kg)	text (number_1dp)
18	demographics_complete	Section Header: *Form Status*<br>Complete?	dropdown<br>0 Incomplete<br>1 Unverified<br>2 Complete

Instrument: **Cardiac Lesion** (cardiac_lesion) Collapse

19	primary_lesion	Which best describes the primary congenital heart disease lesion?	dropdown, Required<br>1 Simple shunt lesions (e.g., ASD, VSD, PAPVR, PDA), repaired/small without sequelae<br>2 Simple shunt lesions with Eisenmenger syndrome<br>3 Simple shunt lesion with clinical sequelae (e.g., PH, arrhythmia, CHF)<br>4 AV septal defect (include isolated primum ASD or inlet VSD in above categories)<br>5 Left-sided obstructive lesions<br>6 Valvar pulmonary stenosis<br>7 D-TGA or physiologically corrected TGA (systemic right ventricle)<br>8 D-TGA s/p arterial switch (systemic left ventricle)<br>9 Tetralogy of Fallot or DORV or pulmonary atresia<br>10 Single ventricle (SV) Fontan-spectrum<br>11 Complex or SV unrepaired cyanotic (e.g., ToF/PA/MAPCA, mixing lesions s/p palliation)<br>12 Ebstein/Uhl anomaly<br>13 Miscellaneous/other<br>14 Heterotaxy spectrum
20	primary_lesion_other<br>Show the field ONLY if:<br>[primary_lesion] = '13'	Describe "other":	notes
21	primary_lesion_descp	Describe CHD lesion.	notes

22	current_physiology	Which best describes the current physiology after intervention?	dropdown<br>1 | Atrial septal defect<br>2 | Ventricular septal defect<br>3 | Atrioventricular septal defect<br>4 | Eisenmenger physiology<br>5 | Left sided valve/outflow tract disease [without coarctation]<br>6 | Valvar pulmonary stenosis<br>7 | Coarctation<br>8 | Tetralogy of Fallot or DORV<br>9 | Pulmonary atresia with intact vent septum<br>10 | Mustard or Senning (atrial switch for TGA)<br>11 | Jatene (arterial switch for TGA)<br>12 | Rastelli procedure for TGA<br>13 | Congenitally corrected TGA<br>14 | Ebstein anomaly<br>15 | Fontan<br>16 | Complex cyanotic (e.g., unrepaired double-inlet ventricle)<br>17 | Other
23	surgical_intervention	Section Header: *Cardiac Procedures. Note that surgical interventions are differentiated from catheter interventions and catheter observations.*<br>Has the patient had surgical intervention for underlying congenital heart disease?	yesno<br>1 | Yes<br>0 | No
24	surg_intervention_amt<br>Show the field ONLY if: [surgical_intervention] ="1"	How many surgical interventions?	dropdown<br>1 | 1<br>2 | 2<br>3 | 3 or more<br>444 | Missing
25	cath_intervention	Has the patient had a catheter intervention procedure for underlying congenital heart disease?	dropdown<br>1 | Yes<br>2 | No<br>3 | Missing
26	cath_observation	Has the patient had a catheter observation procedure for underlying congenital heart disease?	dropdown<br>1 | Yes<br>0 | No<br>444 | Missing
27	nyha	NYHA Functional Class (at most recent visit, or last known record)	dropdown<br>1 | I<br>2 | II<br>3 | III<br>4 | IV<br>444 | Missing
28	pacemaker	Does the patient have a pacemaker?	yesno<br>1 | Yes<br>0 | No
29	icd	Does the patient have an ICD (implantable cardioverter defibrillator)?	yesno<br>1 | Yes<br>0 | No
30	bio_children	Section Header: *Family History*<br>Does patient have biological children?	dropdown<br>1 | Yes<br>0 | No<br>444 | Missing

31	bio_childrenamt<br><br>Show the field ONLY if:<br>[bio_children]="1"	How many biological children?	text (integer)
32	fhx_chd	Does the patient have any family history of congenital heart disease, including biological children?	dropdown<br>1 Yes<br>0 No<br>444 Missing
33	fhx_all<br><br>Show the field ONLY if:<br>[fhx_chd]=1	Check which blood relatives have CHD.	checkbox<br>1 fhx_all___1 Mother<br>2 fhx_all___2 Father<br>3 fhx_all___3 Sibling(s)<br>4 fhx_all___4 Grandparent(s)<br>5 fhx_all___5 Aunt(s)<br>6 fhx_all___6 Uncle(s)<br>7 fhx_all___7 First cousin(s)<br>8 fhx_all___8 Child<br>9 fhx_all___9 Other<br>444 fhx_all___444 Missing
34	fhx_sib<br><br>Show the field ONLY if:<br>[fhx_all(3)] = '1'	How many siblings have CHD?	text (integer, Min: 1, Max: 99)
35	fhx_grandparent<br><br>Show the field ONLY if:<br>[fhx_all(4)] = '1'	How many grandparents have CHD?	text (integer, Min: 1, Max: 4)
36	fhx_aunt<br><br>Show the field ONLY if:<br>[fhx_all(5)] = '1'	How many aunts have CHD?	text (integer, Min: 1, Max: 99)
37	fhx_uncle<br><br>Show the field ONLY if:<br>[fhx_all(6)] = '1'	How many uncles have CHD?	text (integer, Min: 1, Max: 99)
38	fhx_cousin<br><br>Show the field ONLY if:<br>[fhx_all(7)] = '1'	How many first cousins have CHD?	text (integer, Min: 1, Max: 99)
39	fhx_child<br><br>Show the field ONLY if:<br>[fhx_all(8)] = '1'	How many biological children have CHD?	text (integer, Min: 1, Max: 99)
40	fhx_other<br><br>Show the field ONLY if:<br>[fhx_all(9)] = '1'	How many "other" relatives have CHD?	text (integer, Min: 1, Max: 99)
41	genetics_referral	Section Header: *Genetics Referral Status*<br>Has the patient had a referral to clinical genetics?	yesno<br>1 Yes<br>0 No
42	dov_genetics<br><br>Show the field ONLY if:<br>[genetics_referral]="1"	Date of most recent genetics visit<br>*MM-DD-YYYY*	text (date_mdy)
43	genetics_referralstatus<br><br>Show the field ONLY if:<br>[genetics_referral]="1"	What is the status of the genetics referral?	dropdown<br>1 referral made, pending appointment<br>2 consultation completed by geneticist<br>3 consultation completed by genetic counselor<br>4 consultation completed by geneticist and genetic counselor<br>444 missing
44	cardiac_lesion_complete	Section Header: *Form Status*<br>Complete?	dropdown<br>0 Incomplete<br>1 Unverified<br>2 Complete

Instrument: **CHD Family History - Detailed** (chd_family_history_detailed)

45	no_fhx_chd	Check this box if there is no family history of CHD:	radio<br>1 No family history of CHD
46	fhx_chd_relative	Which family member with CHD is being described?	dropdown<br>1 Mother<br>2 Father<br>3 Sibling<br>4 Grandparent<br>5 Aunt<br>6 uncle<br>7 First cousin<br>8 Child<br>9 Other
47	fhx_relativeinfo	No other information regarding this relative is available.	truefalse<br>1 True<br>0 False
48	fhx_chdinfo<br>Show the field ONLY if:<br>[fhx_relativeinfo]="0"	Describe CHD lesion here.	text
49	fhx_ec<br>Show the field ONLY if:<br>[fhx_relativeinfo]=0	Does this person have extracardiac comorbidities, structural malformations, or neurodevelopmental disabilities?	dropdown<br>0 No<br>1 Yes<br>444 Missing
50	fhx_ecdetails<br>Show the field ONLY if:<br>[fhx_relativeinfo]="0" and [fhx_ec]="1"	Describe extracardiac comorbidities, structural malformations, or neurodevelopmental disabilities here.	text
51	fhx_echo<br>Show the field ONLY if:<br>[fhx_relativeinfo]=0	Has this relative had a normal echo or fetal echo?	dropdown<br>0 No<br>1 Yes<br>444 Missing
52	fhx_notes	Other notes regarding family member's CHD or medical history:	notes
53	chd_family_history_detailed_complete	Section Header: *Form Status*<br>Complete?	dropdown<br>0 Incomplete<br>1 Unverified<br>2 Complete

Instrument: **Acquired Comorbidities** (acquired_comorbidities)

54	comorb_cardio	Section Header: *Acquired/ND Comorbidities*<br>Cardiovascular (check all that apply)	checkbox<br>1 | comorb_cardio___1 | Atrial fibrillation (excluding isolated post-operative arrhythmia)<br>2 | comorb_cardio___2 | Atrial flutter/IART<br>3 | comorb_cardio___3 | AV nodal reentrant tachycardia<br>4 | comorb_cardio___4 | Wolf-Parkinson-White<br>5 | comorb_cardio___5 | Non-sustained VT<br>6 | comorb_cardio___6 | Sustained VT or ventricular fibrillation/sudden cardiac death<br>7 | comorb_cardio___7 | Systemic hypertension<br>8 | comorb_cardio___8 | Hyperlipidemia (or dyslipidemia)<br>9 | comorb_cardio___9 | Metabolic syndrome<br>10 | comorb_cardio___10 | Pulmonary hypertension<br>11 | comorb_cardio___11 | Congestive heart failure<br>12 | comorb_cardio___12 | Deep venous thrombosis<br>13 | comorb_cardio___13 | Pulmonary thromboembolism<br>15 | comorb_cardio___15 | Coronary artery disease<br>16 | comorb_cardio___16 | Atherosclerotic coronary artery disease<br>17 | comorb_cardio___17 | Endocarditis<br>14 | comorb_cardio___14 | Other<br>18 | comorb_cardio___18 | None of the above<br>444 | comorb_cardio___444 | Missing
55	comorb_cardio_other<br>Show the field ONLY if:<br>[comorb_cardio(14)] = '1'	Please describe "other":	text
56	comorb_rheum	Rheumatology/Orthopedic (check all that apply)	checkbox<br>1 | comorb_rheum___1 | Gout<br>2 | comorb_rheum___2 | Osteoporosis<br>3 | comorb_rheum___3 | Osteoarthritis (including prior joint replacement)<br>4 | comorb_rheum___4 | Fibromyalgia<br>5 | comorb_rheum___5 | Chronic back pain<br>6 | comorb_rheum___6 | Lupus (SLE)<br>7 | comorb_rheum___7 | Rheumatoid arthritis<br>8 | comorb_rheum___8 | Scleroderma (systemic sclerosis)<br>9 | comorb_rheum___9 | Mixed connective tissue disease<br>10 | comorb_rheum___10 | Psoriasis<br>11 | comorb_rheum___11 | Ankylosing spondylitis<br>12 | comorb_rheum___12 | Scoliosis, moderate+ or s/p surgery<br>13 | comorb_rheum___13 | Carpal tunnel<br>14 | comorb_rheum___14 | Hip fracture<br>15 | comorb_rheum___15 | Spinal stenosis<br>16 | comorb_rheum___16 | Other<br>17 | comorb_rheum___17 | None of the above<br>444 | comorb_rheum___444 | Missing
57	comorb_rheum_other<br>Show the field ONLY if:<br>[comorb_rheum(16)] = '1'	Please describe "other":	text

58	comorb_psych	Psychiatric (check all that apply)	checkbox<br>1 | comorb_psych___1 | Depression<br>2 | comorb_psych___2 | Anxiety<br>3 | comorb_psych___3 | ADHD<br>4 | comorb_psych___4 | Obsessive compulsive disorder<br>5 | comorb_psych___5 | Cognitive impairment/developmental delay<br>6 | comorb_psych___6 | Post-traumatic stress disorder<br>7 | comorb_psych___7 | Schizophrenia<br>8 | comorb_psych___8 | Bipolar disorder<br>9 | comorb_psych___9 | Dementia<br>10 | comorb_psych___10 | Personality disorder<br>11 | comorb_psych___11 | Autism spectrum disorder<br>12 | comorb_psych___12 | Other<br>13 | comorb_psych___13 | None of the above<br>444 | comorb_psych___444 | Missing
59	comorb_psych_other<br>Show the field ONLY if:<br>[comorb_psych(12)] = '1'	Please describe "other":	text
60	comorb_gi	GI (check all that apply)	checkbox<br>1 | comorb_gi___1 | Cirrhosis<br>2 | comorb_gi___2 | Hepatocellular carcinoma<br>3 | comorb_gi___3 | Ascites<br>4 | comorb_gi___4 | Splenomegaly<br>5 | comorb_gi___5 | Hepatitis C<br>6 | comorb_gi___6 | Hepatitis B<br>7 | comorb_gi___7 | Gallbladder disease<br>8 | comorb_gi___8 | GERD or esophagitis<br>9 | comorb_gi___9 | Irritable bowel syndrome<br>10 | comorb_gi___10 | Gastric or duodenal ulcers<br>11 | comorb_gi___11 | Inflammatory bowel syndrome, Crohn's disease<br>12 | comorb_gi___12 | Inflammatory bowel syndrome, ulcerative colitis<br>13 | comorb_gi___13 | Gilbert's disease<br>14 | comorb_gi___14 | Gluten enteropathy/celiac disease<br>15 | comorb_gi___15 | Other<br>16 | comorb_gi___16 | None of the above<br>444 | comorb_gi___444 | Missing
61	comorb_gi_other<br>Show the field ONLY if:<br>[comorb_gi(15)] = '1'	Please describe "other":	text

62	comorb_endorepro	Endocrine/Reproductive (check all that apply)	checkbox<br>1 | comorb_endorepro___1 | Hypothyroidism (Hashimoto'[illegible] disease)<br>2 | comorb_endorepro___2 | Hyperthyroidism (Grave's dis[illegible]<br>3 | comorb_endorepro___3 | Type 2 diabetes mellitus (mo[illegible] common)<br>4 | comorb_endorepro___4 | Type 1 diabetes mellitus (juv[illegible]<br>5 | comorb_endorepro___5 | Paraganglioma/pheochromo[illegible]<br>6 | comorb_endorepro___6 | Hypoparathyroidism<br>7 | comorb_endorepro___7 | PCOS (Polycystic ovary syndr[illegible]<br>8 | comorb_endorepro___8 | Endometriosis<br>9 | comorb_endorepro___9 | Other<br>10 | comorb_endorepro___10 | None of the above<br>444 | comorb_endorepro___444 | Missing
63	comorb_endorepro_other<br>Show the field ONLY if:<br>[comorb_endorepro(9)] = '1'	Please describe "other":	text
64	comorb_pulm	Pulmonary (check all that apply)	checkbox<br>1 | comorb_pulm___1 | Obstructive sleep apnea<br>2 | comorb_pulm___2 | Asthma<br>3 | comorb_pulm___3 | COPD or emphysema<br>4 | comorb_pulm___4 | Restrictive lung disease<br>5 | comorb_pulm___5 | Interstitial lung disease<br>6 | comorb_pulm___6 | Bronchiectasis<br>7 | comorb_pulm___7 | Sequestration<br>8 | comorb_pulm___8 | Diaphragmatic dysfunction<br>9 | comorb_pulm___9 | Other<br>10 | comorb_pulm___10 | None of the above<br>444 | comorb_pulm___444 | Missing
65	comorb_pulm_other<br>Show the field ONLY if:<br>[comorb_pulm(9)] = '1'	Please describe "other":	text
66	comorb_neuro	Neurology (check all that apply)	checkbox<br>1 | comorb_neuro___1 | Epilepsy/seizure disorder<br>2 | comorb_neuro___2 | Transient ischemia event<br>3 | comorb_neuro___3 | Cerebrovascular accident/stroke<br>4 | comorb_neuro___4 | Migraine headache<br>5 | comorb_neuro___5 | Dementia<br>6 | comorb_neuro___6 | Parkinson's<br>7 | comorb_neuro___7 | Sleep problem (other than sleep apnea)<br>8 | comorb_neuro___8 | Multiple sclerosis<br>9 | comorb_neuro___9 | Peripheral neuropathy<br>10 | comorb_neuro___10 | Other<br>11 | comorb_neuro___11 | None of the above<br>444 | comorb_neuro___444 | Missing
67	comorb_neuro_other<br>Show the field ONLY if:<br>[comorb_neuro(10)] = '1'	Please describe "other":	text

68	comorb_onco	Cancer (check all that apply)	checkbox<br>1 | comorb_onco___1 | Breast<br>2 | comorb_onco___2 | Colon or rectal<br>3 | comorb_onco___3 | Endometrial<br>4 | comorb_onco___4 | Kidney (renal cell)<br>5 | comorb_onco___5 | Leukemia<br>6 | comorb_onco___6 | Liver (hepatocellular)<br>7 | comorb_onco___7 | Lung<br>8 | comorb_onco___8 | Lymphoma<br>9 | comorb_onco___9 | Melanoma<br>10 | comorb_onco___10 | Non-melanoma skin<br>11 | comorb_onco___11 | Prostate<br>12 | comorb_onco___12 | Thyroid<br>13 | comorb_onco___13 | Other<br>14 | comorb_onco___14 | None of the above<br>444 | comorb_onco___444 | Missing
69	comorb_onco_other<br>Show the field ONLY if:<br>[comorb_onco(13)] = '1'	Please describe "other":	text
70	comorb_heme	Hematology (check all that apply)	checkbox<br>1 | comorb_heme___1 | Anemia, any<br>2 | comorb_heme___2 | Pernicious anemia/B12 deficiency<br>3 | comorb_heme___3 | Iron deficiency, current/recent < 6mos<br>4 | comorb_heme___4 | Iron deficiency, ever<br>5 | comorb_heme___5 | Anemia, blood loss<br>6 | comorb_heme___6 | Hereditary hemoglobinopathy<br>7 | comorb_heme___7 | Secondary erythrocytosis<br>8 | comorb_heme___8 | Thrombocytopenia<br>9 | comorb_heme___9 | Other<br>10 | comorb_heme___10 | None of the above<br>444 | comorb_heme___444 | Missing
71	comorb_heme_other<br>Show the field ONLY if:<br>[comorb_heme(9)] = '1'	Please describe "other":	text
72	comorb_renal	Renal (check all that apply)	checkbox<br>1 | comorb_renal___1 | Chronic kidney disease (renal insufficiency)<br>2 | comorb_renal___2 | Nephrotic syndrome<br>3 | comorb_renal___3 | Other (including acute renal disease)<br>4 | comorb_renal___4 | None of the above<br>444 | comorb_renal___444 | Missing
73	comorb_renal_other<br>Show the field ONLY if:<br>[comorb_renal(3)] = '1'	Please describe "other":	text
74	comorb_misc	Miscellaneous/Other	text
75	comorb_generalnotes	Notes on Neurodevelopmental or Acquired Comorbidities	text
76	acquired_comorbidities_complete	Section Header: *Form Status*<br>Complete?	dropdown<br>0 | Incomplete<br>1 | Unverified<br>2 | Complete

Instrument: **Congenital Comorbidities** (congenital_comorbidities) Collapse

<table>
<tr><td>77</td><td>congcomorb_all</td><td>Does the patient have congenital malformations in any of the following? (check all the apply)</td><td>checkbox<table>
<tr><td>1</td><td>congcomorb_all___1</td><td>Brain</td></tr>
<tr><td>2</td><td>congcomorb_all___2</td><td>Ear, nose, throat, mouth</td></tr>
<tr><td>3</td><td>congcomorb_all___3</td><td>Eye</td></tr>
<tr><td>4</td><td>congcomorb_all___4</td><td>Neck and chest</td></tr>
<tr><td>5</td><td>congcomorb_all___5</td><td>Lung</td></tr>
<tr><td>6</td><td>congcomorb_all___6</td><td>Endocrine</td></tr>
<tr><td>7</td><td>congcomorb_all___7</td><td>GI and abdominal wall</td></tr>
<tr><td>8</td><td>congcomorb_all___8</td><td>Kidney</td></tr>
<tr><td>9</td><td>congcomorb_all___9</td><td>Spleen</td></tr>
<tr><td>10</td><td>congcomorb_all___10</td><td>Pancreas - annular pancreas</td></tr>
<tr><td>11</td><td>congcomorb_all___11</td><td>Liver/Gall bladder</td></tr>
<tr><td>12</td><td>congcomorb_all___12</td><td>Anus - Imperforate anus or anal atresia</td></tr>
<tr><td>13</td><td>congcomorb_all___13</td><td>GU</td></tr>
<tr><td>14</td><td>congcomorb_all___14</td><td>Skeletal/limb</td></tr>
<tr><td>16</td><td>congcomorb_all___16</td><td>Lymphatic - lymphatic dysplasia</td></tr>
<tr><td>17</td><td>congcomorb_all___17</td><td>Skin</td></tr>
<tr><td>18</td><td>congcomorb_all___18</td><td>arteriovenous malformation</td></tr>
<tr><td>19</td><td>congcomorb_all___19</td><td>Craniofacial dysmorphism</td></tr>
<tr><td>20</td><td>congcomorb_all___20</td><td>None of the above</td></tr>
<tr><td>21</td><td>congcomorb_all___21</td><td>Other</td></tr>
<tr><td>444</td><td>congcomorb_all___444</td><td>Missing</td></tr>
</table></td></tr>
<tr><td>78</td><td>congcomorb_brain
Show the field ONLY if:
[congcomorb_all(1)] = '1'</td><td>Brain congenital malformations</td><td>checkbox<table>
<tr><td>1</td><td>congcomorb_brain___1</td><td>Agenesi corpus [illegible]</td></tr>
<tr><td>2</td><td>congcomorb_brain___2</td><td>Hydroce[illegible]</td></tr>
<tr><td>3</td><td>congcomorb_brain___3</td><td>Cerebell[illegible] hypopla[illegible] posterio[illegible] abn)</td></tr>
<tr><td>4</td><td>congcomorb_brain___4</td><td>Dandy w[illegible] malform[illegible]</td></tr>
<tr><td>5</td><td>congcomorb_brain___5</td><td>holopro[illegible]</td></tr>
<tr><td>molymicrogyri</td><td>congcomorb_brain___molymicrogyri</td><td>pachygr[illegible]</td></tr>
<tr><td>6</td><td>congcomorb_brain___6</td><td>lissence[illegible]</td></tr>
<tr><td>7</td><td>congcomorb_brain___7</td><td>Heteroto[illegible] (other m[illegible] abn)</td></tr>
<tr><td>8</td><td>congcomorb_brain___8</td><td>Immatu[illegible] grey/wh[illegible] differen[illegible] other co[illegible]</td></tr>
<tr><td>9</td><td>congcomorb_brain___9</td><td>Neural t[illegible] defects</td></tr>
<tr><td>10</td><td>congcomorb_brain___10</td><td>other</td></tr>
<tr><td>443</td><td>congcomorb_brain___443</td><td>Not spe[illegible]</td></tr>
</table></td></tr>
<tr><td>79</td><td>congcomorb_brain_other
Show the field ONLY if:
[congcomorb_brain(10)] = '1'</td><td>List "other" Brain congenital malformation</td><td>text</td></tr>
</table>

80	congcomorb_ntd<br>Show the field ONLY if:<br>[congcomorb_brain(9)] = '1'	Type of neural tube defect	checkbox<br>1 congcomorb_ntd___1 Encephalocele<br>2 congcomorb_ntd___2 Myelomeningocele<br>3 congcomorb_ntd___3 Arnold Chiari malformations<br>4 congcomorb_ntd___4 Spina bifida<br>5 congcomorb_ntd___5 other<br>443 congcomorb_ntd___443 Not specified
81	congcomorb_ntd_other<br>Show the field ONLY if:<br>[congcomorb_ntd(5)] = '1'	List "other" Type of neural tube defect	text
82	congcomorb_ent<br>Show the field ONLY if:<br>[congcomorb_all(2)] = '1'	ENT and mouth congenital malformations	checkbox<br>1 congcomorb_ent___1 Ears<br>2 congcomorb_ent___2 Nose - choanal atresia or stenosis<br>3 congcomorb_ent___3 Palate<br>4 congcomorb_ent___4 Skull - carniosynostosis<br>5 congcomorb_ent___5 Neck - congenital torticollis<br>6 congcomorb_ent___6 Tracheoesphageal atresia<br>7 congcomorb_ent___7 Tracheosesphageal fistula<br>8 congcomorb_ent___8 Laryngomalacia/tracheomalacia<br>9 congcomorb_ent___9 macroglossia<br>10 congcomorb_ent___10 micrognathia<br>11 congcomorb_ent___11 bifid uvula<br>12 congcomorb_ent___12 other<br>443 congcomorb_ent___443 Not specified
83	congcomorb_ent_other<br>Show the field ONLY if:<br>[congcomorb_ent(12)] = '1'	List "other" ENT and mouth congenital malformations	text
84	congcomorb_ent_ear<br>Show the field ONLY if:<br>[congcomorb_ent(1)] = '1'	Type of ear abnormality	checkbox<br>1 congcomorb_ent_ear___1 Microtia<br>2 congcomorb_ent_ear___2 Abnormalities of the semicircular canals<br>3 congcomorb_ent_ear___3 Absent/Atresia of the canal<br>4 congcomorb_ent_ear___4 ear pit<br>5 congcomorb_ent_ear___5 ear tag<br>6 congcomorb_ent_ear___6 hearing loss or congenital deafness<br>7 congcomorb_ent_ear___7 low set ears<br>8 congcomorb_ent_ear___8 other<br>443 congcomorb_ent_ear___443 Not specified
85	congcomorb_ent_earother<br>Show the field ONLY if:<br>[congcomorb_ent_ear(8)] = '1'	List "other" type of ear abnormality	text
86	congcomorb_ent_palate<br>Show the field ONLY if:<br>[congcomorb_ent(3)] = '1'	Type of palate abnormality	checkbox<br>1 congcomorb_ent_palate___1 Cleft lip<br>2 congcomorb_ent_palate___2 Cleft palate<br>3 congcomorb_ent_palate___3 other<br>443 congcomorb_ent_palate___443 Not specified
87	congcomorb_ent_palateother<br>Show the field ONLY if:<br>[congcomorb_ent_palate(3)] = '1'	List "other" type of palate abnormality	text

<table>
<tr><td>88</td><td>congcomorb_eye
Show the field ONLY if:
[congcomorb_all(3)] = '1'</td><td>Eye congenital malformations and abnormalities</td><td>checkbox<table>
<tr><td>1</td><td>congcomorb_eye___1</td><td>Coloboma</td></tr>
<tr><td>2</td><td>congcomorb_eye___2</td><td>Microphthalmos</td></tr>
<tr><td>3</td><td>congcomorb_eye___3</td><td>Congenital glaucoma</td></tr>
<tr><td>4</td><td>congcomorb_eye___4</td><td>Congenital cataract</td></tr>
<tr><td>5</td><td>congcomorb_eye___5</td><td>Retinal anomaly</td></tr>
<tr><td>6</td><td>congcomorb_eye___6</td><td>Posterior embryotoxon</td></tr>
<tr><td>7</td><td>congcomorb_eye___7</td><td>anophthalmia</td></tr>
<tr><td>8</td><td>congcomorb_eye___8</td><td>congenital blindness</td></tr>
<tr><td>9</td><td>congcomorb_eye___9</td><td>esotropia</td></tr>
<tr><td>10</td><td>congcomorb_eye___10</td><td>epicanthal folds</td></tr>
<tr><td>11</td><td>congcomorb_eye___11</td><td>nystagmus</td></tr>
<tr><td>12</td><td>congcomorb_eye___12</td><td>strabismus</td></tr>
<tr><td>13</td><td>congcomorb_eye___13</td><td>other</td></tr>
</table></td></tr>
<tr><td>89</td><td>congcomorb_eye_other
Show the field ONLY if:
[congcomorb_eye(13)] = '1'</td><td>List "other" eye congenital malformation</td><td>text</td></tr>
<tr><td>90</td><td>congcomorb_chest
Show the field ONLY if:
[congcomorb_all(4)] = '1'</td><td>Chest and neck congenital malformations</td><td>checkbox<table>
<tr><td>1</td><td>congcomorb_chest___1</td><td>Pectus excavatum or carinatum</td></tr>
<tr><td>2</td><td>congcomorb_chest___2</td><td>Split sternum</td></tr>
<tr><td>3</td><td>congcomorb_chest___3</td><td>Anomaly of the clavicles</td></tr>
<tr><td>4</td><td>congcomorb_chest___4</td><td>branchial cleft/cyst</td></tr>
<tr><td>5</td><td>congcomorb_chest___5</td><td>excess nuchal skin</td></tr>
<tr><td>6</td><td>congcomorb_chest___6</td><td>webbed neck</td></tr>
<tr><td>7</td><td>congcomorb_chest___7</td><td>supernumerary nipples</td></tr>
<tr><td>8</td><td>congcomorb_chest___8</td><td>other</td></tr>
</table></td></tr>
<tr><td>91</td><td>congcomorb_chest_other
Show the field ONLY if:
[congcomorb_chest(8)] = '1'</td><td>List "other" chest and neck congenital malformation</td><td>text</td></tr>
<tr><td>92</td><td>congcomorb_lung
Show the field ONLY if:
[congcomorb_all(5)] = '1'</td><td>Lung congenital malformations</td><td>checkbox<table>
<tr><td>1</td><td>congcomorb_lung___1</td><td>Isomerisom</td></tr>
<tr><td>2</td><td>congcomorb_lung___2</td><td>Scimitar syndrome</td></tr>
<tr><td>3</td><td>congcomorb_lung___3</td><td>Cystic adenomatoid malformation</td></tr>
<tr><td>4</td><td>congcomorb_lung___4</td><td>Pulmonary agenesis or hypoplasia</td></tr>
<tr><td>5</td><td>congcomorb_lung___5</td><td>congenital diaphragmatic hernia</td></tr>
<tr><td>6</td><td>congcomorb_lung___6</td><td>other</td></tr>
<tr><td>443</td><td>congcomorb_lung___443</td><td>Not specified</td></tr>
</table></td></tr>
<tr><td>93</td><td>congcomorb_lung_other
Show the field ONLY if:
[congcomorb_lung(6)] = '1'</td><td>List "other" lung congenital malformation</td><td>text</td></tr>
<tr><td>94</td><td>congcomorb_endo
Show the field ONLY if:
[congcomorb_all(6)] = '1'</td><td>Endocrine abnormalities and malformations</td><td>checkbox<table>
<tr><td>1</td><td>congcomorb_endo___1</td><td>thyroid agenesis</td></tr>
<tr><td>2</td><td>congcomorb_endo___2</td><td>adrenal dysgenesis</td></tr>
<tr><td>3</td><td>congcomorb_endo___3</td><td>other</td></tr>
<tr><td>443</td><td>congcomorb_endo___443</td><td>Not specified</td></tr>
</table></td></tr>
<tr><td>95</td><td>congcomorb_endo_other
Show the field ONLY if:
[congcomorb_endo(3)] = '1'</td><td>List "other" endocrine abnormality or malformation</td><td>text</td></tr>
</table>

<table>
<tr><td>96</td><td>congcomorb_gi

Show the field ONLY if:
[congcomorb_all(7)] = '1'</td><td>GI and abdominal wall malformations</td><td>checkbox<table><tr><td>1</td><td>congcomorb_gi___1</td><td>Duodenal atresia</td></tr><tr><td>2</td><td>congcomorb_gi___2</td><td>Microcolon</td></tr><tr><td>3</td><td>congcomorb_gi___3</td><td>Hirschprungs</td></tr><tr><td>4</td><td>congcomorb_gi___4</td><td>Gastroschisis</td></tr><tr><td>5</td><td>congcomorb_gi___5</td><td>Omphalocele</td></tr><tr><td>6</td><td>congcomorb_gi___6</td><td>Prune belly</td></tr><tr><td>7</td><td>congcomorb_gi___7</td><td>Esophageal atresia</td></tr><tr><td>8</td><td>congcomorb_gi___8</td><td>Intestinal atresia</td></tr><tr><td>9</td><td>congcomorb_gi___9</td><td>Malrotation</td></tr><tr><td>10</td><td>congcomorb_gi___10</td><td>Inguinal or abdominal hernia</td></tr><tr><td>11</td><td>congcomorb_gi___11</td><td>pyloric stenosis</td></tr><tr><td>12</td><td>congcomorb_gi___12</td><td>other</td></tr><tr><td>443</td><td>congcomorb_gi___443</td><td>Not specified</td></tr></table></td></tr>
<tr><td>97</td><td>congcomorb_gi_other

Show the field ONLY if:
[congcomorb_gi(12)] = '1'</td><td>List "other' GI and abdominal wall malformations</td><td>text</td></tr>
<tr><td>98</td><td>congcomorb_renal

Show the field ONLY if:
[congcomorb_all(8)] = '1'</td><td>Kidney congenital malformations</td><td>checkbox<table><tr><td>1</td><td>congcomorb_renal___1</td><td>Unilaterial kidney</td></tr><tr><td>2</td><td>congcomorb_renal___2</td><td>Single kidney</td></tr><tr><td>3</td><td>congcomorb_renal___3</td><td>Horsehoe kidney(s)</td></tr><tr><td>4</td><td>congcomorb_renal___4</td><td>Renal agenesis</td></tr><tr><td>5</td><td>congcomorb_renal___5</td><td>Multicystic kidneys or dysplastic kidney(s)</td></tr><tr><td>6</td><td>congcomorb_renal___6</td><td>Duplicated collecting system</td></tr><tr><td>7</td><td>congcomorb_renal___7</td><td>other</td></tr><tr><td>443</td><td>congcomorb_renal___443</td><td>Not specified</td></tr></table></td></tr>
<tr><td>99</td><td>congcomorb_renal_other

Show the field ONLY if:
[congcomorb_renal(7)] = '1'</td><td>List "other" kidney congenital malformation</td><td>text</td></tr>
<tr><td>100</td><td>congcomorb_spleen

Show the field ONLY if:
[congcomorb_all(9)] = '1'</td><td>Spleen congenital malformations</td><td>checkbox<table><tr><td>1</td><td>congcomorb_spleen___1</td><td>Asplenia</td></tr><tr><td>2</td><td>congcomorb_spleen___2</td><td>Polysplenia</td></tr><tr><td>3</td><td>congcomorb_spleen___3</td><td>other</td></tr><tr><td>443</td><td>congcomorb_spleen___443</td><td>Not specified</td></tr></table></td></tr>
<tr><td>101</td><td>congcomorb_spleen_other

Show the field ONLY if:
[congcomorb_spleen(3)] = '1'</td><td>List "other" spleen congenital malformation</td><td>text</td></tr>
<tr><td>102</td><td>congcomorb_liver

Show the field ONLY if:
[congcomorb_all(11)] = '1'</td><td>Liver/Gall bladder congenital malformations</td><td>checkbox<table><tr><td>1</td><td>congcomorb_liver___1</td><td>Biliary atresia</td></tr><tr><td>2</td><td>congcomorb_liver___2</td><td>Choledochal cyst</td></tr><tr><td>3</td><td>congcomorb_liver___3</td><td>Midline liver</td></tr><tr><td>4</td><td>congcomorb_liver___4</td><td>other</td></tr><tr><td>443</td><td>congcomorb_liver___443</td><td>Not specified</td></tr></table></td></tr>
<tr><td>103</td><td>congcomorb_liver_other

Show the field ONLY if:
[congcomorb_liver(4)] = '1'</td><td>List "other" liver/gall bladder congenital malformations</td><td>text</td></tr>
</table>

104	congcomorb_gu<br><br>Show the field ONLY if:<br>[congcomorb_all(13)] = '1'	GU congenital malformations	checkbox<br>1 congcomorb_gu___1 Ambiguous genitalia<br>2 congcomorb_gu___2 Bilaterial cryptorchidism<br>3 congcomorb_gu___3 Unilateral cryptorchidism<br>4 congcomorb_gu___4 Hypospadias<br>5 congcomorb_gu___5 Chordee<br>6 congcomorb_gu___6 Vaginal agenesis/atresia/vaginal septum<br>7 congcomorb_gu___7 Ureteral/uterine/vaginal duplication<br>8 congcomorb_gu___8 Ovarian agenesis/dysgenesis<br>9 congcomorb_gu___9 Bladder exstrophy<br>10 congcomorb_gu___10 anteriorly placed anus<br>11 congcomorb_gu___11 bifid scrotum<br>12 congcomorb_gu___12 imperforate/atretic anus<br>13 congcomorb_gu___13 micropenis<br>14 congcomorb_gu___14 undescended testis<br>15 congcomorb_gu___15 other<br>443 congcomorb_gu___443 Not specified
105	congcomorb_gu_other<br><br>Show the field ONLY if:<br>[congcomorb_gu(15)] = '1'	List "other" GU congenital malformations	text
106	congcomorb_skeletal<br><br>Show the field ONLY if:<br>[congcomorb_all(14)] = '1'	Skeletal/limb congenital malformations	checkbox<br>1 congcomorb_skeletal___1 Extra rib(s)<br>2 congcomorb_skeletal___2 Rib fusion(s)<br>3 congcomorb_skeletal___3 Hemivertebrae<br>4 congcomorb_skeletal___4 Absent vertebrae<br>5 congcomorb_skeletal___5 Butterfly vertebrae<br>6 congcomorb_skeletal___6 polydactyly<br>7 congcomorb_skeletal___7 Syndactyly<br>8 congcomorb_skeletal___8 Limb reduction defects<br>9 congcomorb_skeletal___9 Arthrogryposis<br>10 congcomorb_skeletal___10 Clubfoot<br>11 congcomorb_skeletal___11 Congenital hip dislocation<br>12 congcomorb_skeletal___12 Absent radius<br>13 congcomorb_skeletal___13 Scoliosis, 20 degrees or greater<br>14 congcomorb_skeletal___14 Other<br>443 congcomorb_skeletal___443 Not specified
107	congcomorb_skeletal_other<br><br>Show the field ONLY if:<br>[congcomorb_skeletal(14)] = '1'	List "other" skeletal/limb congenital malformation	text
108	congcomorb_skin<br><br>Show the field ONLY if:<br>[congcomorb_all(17)] = '1'	Skin congenital malformations	checkbox<br>1 congcomorb_skin___1 Hemangioma<br>2 congcomorb_skin___2 Aplasia cutis congenital<br>3 congcomorb_skin___3 Ectodermal dysplasia<br>4 congcomorb_skin___4 cafe au lait spots<br>5 congcomorb_skin___5 hyperpigmented lesions<br>6 congcomorb_skin___6 hypopigmented lesions<br>7 congcomorb_skin___7 port wine spots<br>8 congcomorb_skin___8 telangiectasia<br>9 congcomorb_skin___9 other<br>444 congcomorb_skin___444 Not specified

109	congcomorb_skin_other<br>Show the field ONLY if:<br>[congcomorb_skin(9)] = '1'	List "other" skin congenital malformations	text
110	congcomorb_ent_cranf<br>Show the field ONLY if:<br>[congcomorb_all(19)] = '1'	Craniofacial dysmorphism	checkbox<br>1 congcomorb_ent_cranf___1 macrocephaly<br>2 congcomorb_ent_cranf___2 microcephaly<br>3 congcomorb_ent_cranf___3 craniosynostosis<br>4 congcomorb_ent_cranf___4 other<br>443 congcomorb_ent_cranf___443 Not specified
111	congcomorb_ent_cranfother<br>Show the field ONLY if:<br>[congcomorb_ent_cranf(4)] = '1'	List "other" craniofacial dysmorphism	text
112	congcomorb_cranio<br>Show the field ONLY if:<br>[congcomorb_ent_cranf(3)] = '1'	Type of craniosynostosis	checkbox<br>1 congcomorb_cranio___1 sagittal<br>2 congcomorb_cranio___2 metopic<br>3 congcomorb_cranio___3 coronal<br>4 congcomorb_cranio___4 other<br>443 congcomorb_cranio___443 Not specified
113	congcomorb_cranio_other<br>Show the field ONLY if:<br>[congcomorb_cranio(4)] = '1'	List "other" type of craniosynostosis	text
114	congcomorb_other<br>Show the field ONLY if:<br>[congcomorb_all(21)] = '1'	List "other" congenital abnormality, if none of the above categories apply.	text
115	congcomorb_generalnotes	Notes on Congenital Comorbidities	notes
116	congenital_comorbidities_complete	Section Header: *Form Status*<br>Complete?	dropdown<br>0 Incomplete<br>1 Unverified<br>2 Complete

Instrument: **Genetics** (genetics)

Collapse

117	syndrome	Section Header: *Syndromes*<br>Does the patient have a genetic syndromic diagnosis?	radio<br>1 Yes<br>0 No<br>444 Missing
118	syndrome_genetic<br>Show the field ONLY if:<br>[syndrome]="1"	Select a genetic syndrome diagnosis.	dropdown<br>1 Down<br>2 22q11 (DiGeorge, CATCH 22, VCF)<br>3 Heterotaxy syndrome<br>4 Turner<br>5 Noonan<br>6 Williams<br>7 Goldenhar<br>8 Holt-Oram<br>9 Alagille<br>10 Ellis-Van Creveld<br>11 VACTERL<br>12 CHARGE<br>13 Other<br>443 Not specified
119	syndrome_geneticother<br>Show the field ONLY if:<br>[syndrome_genetic]="13"	List "other" genetic syndrome	text

120	prenatalexposure<br><br>Show the field ONLY if:<br>[syndrome]="0"	Has the patient had a prenatal exposure?	radio<br>1 Yes<br>0 No<br>2 Unknown/missing
121	prenatalexposure_dx<br><br>Show the field ONLY if:<br>[prenatalexposure]="1"	What prenatal exposure occurred?	dropdown<br>1 Fetal alcohol spectrum disorder<br>2 Maternal PKU syndrome<br>3 Cytomegalovirus<br>4 Other prenatal exposure<br>443 Not specified
122	prenatalexposure_dxother<br><br>Show the field ONLY if:<br>[prenatalexposure_dx]="4"	List "other" prenatal exposure	text
123	syndrome_notes	Other notes regarding genetic diagnosis:	notes
124	clinmolec_dx<br><br>Show the field ONLY if:<br>[syndrome]="1"	Section Header: *Genetic Testing*<br>Is the genetic diagnosis clinical or molecular?	radio<br>1 Clinical<br>2 Molecular<br>444 Missing
125	genetictest	Has the patient had genetic testing performed?	radio<br>1 Yes<br>0 No<br>444 Missing
126	genetictest_amt<br><br>Show the field ONLY if:<br>[genetictest] = '1'	How many genetic tests?	text (integer, Min: 1, Max: 99)
127	genetics_complete	Section Header: *Form Status*<br>Complete?	dropdown<br>0 Incomplete<br>1 Unverified<br>2 Complete

Instrument: **Genetic Testing - Detailed** (genetic_testing_detailed) Collapse

128	genetic_testing_none	If there are no details about genetic testing, please indicate reason:	dropdown<br>1 No genetic testing performed<br>2 Primary report was not available<br>3 Other
129	genetictest_none_reason<br><br>Show the field ONLY if:<br>[genetic_testing_none] = '3'	Please describe "other":	notes
130	genetictest_type	What type of genetic testing?	dropdown<br>1 Karyotype<br>2 Microarray<br>3 multigene panel<br>4 WES/WGS<br>6 Single gene<br>7 FISH<br>8 Other<br>443 Not specified<br>444 missing
131	genetictest_typeother<br><br>Show the field ONLY if:<br>[genetictest_type]="8"	List "other" genetic test type	text

132	genetictest_result	What is the genetic testing result?	dropdown<br>1 PATH/disease-causing<br>2 LPATH<br>3 VUS<br>4 LBEN<br>5 BEN<br>6 normal<br>7 awaiting result<br>8 other<br>444 Missing
133	genetictest_resultother<br>Show the field ONLY if:<br>[genetictest_result]="8"	If result was "other" variant classification, list here:	text
134	genticttest_variant<br>Show the field ONLY if:<br>[genetictest_result] = '1' or [genetictest_result] = '2' or [genetictest_result] = '3' or [genetictest_result] = '4' or [genetictest_result] = '5' or [genetictest_result]="8"	What is the genetic variant identified on genetic testing?	text
135	genetictest_varianttype<br>Show the field ONLY if:<br>[genetictest_result]="1" or [genetictest_result]="2" or [genetictest_result]="3"	If result was PATH, LPATH, or VUS, what type of variant was identified?	dropdown<br>1 single nucleotide point mutation<br>2 Copy number variant<br>3 chromosomal abnormality of a single chromosome<br>4 Polyploidy<br>5 More than one of the above<br>6 Other<br>444 missing
136	genetictest_date	Year genetic testing report issued, or disclosed to patient if no report date is available	text
137	genetictest_notes	Other notes regarding genetic testing:	notes
138	genetic_testing_detailed_complete	Section Header: *Form Status*<br>Complete?	dropdown<br>0 Incomplete<br>1 Unverified<br>2 Complete
